# Whole genome comparative analysis of channel catfish (*Ictalurus punctatus*) with four model fish species

**DOI:** 10.1186/1471-2164-14-780

**Published:** 2013-11-11

**Authors:** Yanliang Jiang, Xiaoyu Gao, Shikai Liu, Yu Zhang, Hong Liu, Fanyue Sun, Lisui Bao, Geoff Waldbieser, Zhanjiang Liu

**Affiliations:** 1 The Fish Molecular Genetics and Biotechnology Laboratory, Department of Fisheries and Allied Aquacultures, Program of Cell and Molecular Biosciences, Aquatic Genomics Unit, 203 Swingle Hall, Auburn University, Auburn, AL 36849, USA; 2 USDA-ARS Catfish Genetics Research Unit, 141 Experiment Station Road, Stoneville, MS 38776, USA

**Keywords:** Catfish, Genome, Comparative mapping, Linkage mapping, Conserved synteny

## Abstract

**Background:**

Comparative mapping is a powerful tool to study evolution of genomes. It allows transfer of genome information from the well-studied model species to non-model species. Catfish is an economically important aquaculture species in United States. A large amount of genome resources have been developed from catfish including genetic linkage maps, physical maps, BAC end sequences (BES), integrated linkage and physical maps using BES-derived markers, physical map contig-specific sequences, and draft genome sequences. Application of such genome resources should allow comparative analysis at the genome scale with several other model fish species.

**Results:**

In this study, we conducted whole genome comparative analysis between channel catfish and four model fish species with fully sequenced genomes, zebrafish, medaka, stickleback and *Tetraodon*. A total of 517 Mb draft genome sequences of catfish were anchored to its genetic linkage map, which accounted for 62% of the total draft genome sequences. Based on the location of homologous genes, homologous chromosomes were determined among catfish and the four model fish species. A large number of conserved syntenic blocks were identified. Analysis of the syntenic relationships between catfish and the four model fishes supported that the catfish genome is most similar to the genome of zebrafish.

**Conclusion:**

The organization of the catfish genome is similar to that of the four teleost species, zebrafish, medaka, stickleback, and *Tetraodon* such that homologous chromosomes can be identified. Within each chromosome, extended syntenic blocks were evident, but the conserved syntenies at the chromosome level involve extensive inter-chromosomal and intra-chromosomal rearrangements. This whole genome comparative map should facilitate the whole genome assembly and annotation in catfish, and will be useful for genomic studies of various other fish species.

## Background

With the advances of next generation sequencing technology, genomic resources are rapidly expanding, even for non-model species. Among teleost species, whole genomes of five model species have been fully sequenced and assembled, including zebrafish (*Danio rerio*) (http://www.ensembl.org), fugu (*Fugu rubripes*) [1], *Tetraodon* (*Tetraodon nigroviridis*) [2], medaka (*Oryzias latipes*) [3,4] and three-spined stickleback (*Gasterosteus aculeatus*) [5]. Among aquaculture fish species, whole genome reference sequence has been only published for Atlantic cod [6], although genomes of many aquaculture species have been or are being sequenced. In recent years, great efforts on generating genomic resources have been made for economically important aquaculture species [7], such as Atlantic salmon [8-11], European sea bass [12-16], tilapia [17-22], rainbow trout [23-28], gilthead sea bream [29,30], and catfish (for reviews, see [31,32]). These genomic resources included expressed sequence tags (ESTs), BAC end sequences, physical maps, genetic linkage maps, and radiation hybrid maps.

In the absence of whole genome sequences for most aquaculture species, comparative genomic analysis is useful. Comparative mapping allows identification of evolutionarily conserved chromosomal regions, i.e., conserved syntenies, which facilitate the understanding of genome organization, rearrangement, duplication and evolution [33-37]. Moreover, the conserved syntenies provide physical evidence for orthologies and genome annotation, which is particularly important when dealing with multi-gene families [38,39]. Comparative genome analysis can also enhance the efficiency for the identification of candidate genes controlling production traits of interest, when coupled with quantitative trait loci (QTL) mapping analysis [7].

Comparative mapping was initially demonstrated by Fujiyama et al. [40] for constructing the human-chimpanzee comparative map using chimpanzee BAC end sequences to hit against human genome sequences. Putative orthologues were identified between these two closely related species. Later on, comparative mapping was extensively performed among mammals, such as the construction of human-cattle [41], human-porcine [42], human-horse [43] and human-sheep [44] comparative maps. High percentage of BLAST hits and/or high level of genome colinearity made the comparative mapping successful [45]. However, whole genome comparative mapping in most teleost species is still limited due to lacking of genomic resources.

Channel catfish, *Ictalurus punctatus*, is the predominant aquaculture species in the United States. To gain understanding of the catfish genome, considerable efforts have been made toward the development of genomic resources, including genetic linkage maps [46-49], large-insert libraries [50,51], physical maps [52,53], BAC end sequences [45,54], a large number of Sanger sequenced ESTs from various tissues and developmental stages [55-59], full length cDNAs [60], RNA-Seq transcriptome assemblies [61,62], and whole genome shotgun sequence reads (unpublished). Such genomic resources provided a foundation for comparative analysis. For instance, Wang et al. [51] utilized catfish BAC end sequences to compare with zebrafish and *Tetraodon* genome, and identified conserved synteny regions in the catfish genome. More recently, Liu et al. [45] conducted comparative mapping analysis by using a large number of BAC end sequences. Genetic linkage map containing type I gene-associated markers was also used for comparative analysis [46]. With next-generation sequencing data, Jiang et al. [63] conducted comparative analysis between an approximately 1 Mb DNA region in catfish genome with other model fish species. Recently, one catfish linkage group (chromosome) was compared with model fish [64]. These studies allowed for identification of conserved syntenies in the catfish genome as compared with other sequenced fish genomes. In these studies, however, only a small number of gene markers or only one chromosome was used for comparative analysis.

To obtain detailed comparative information at the genome level, whole genome comparative analysis is much needed. We report here the whole genome comparative analysis of catfish with four model fish species, zebrafish, medaka, *Tetraodon* and stickleback, utilizing all currently available catfish genomic resources. With the whole genome comparative mapping, homologous chromosomes were identified and a large number of conserved syntenies were identified.

## Results

### Identification of genomic sequences mapped on the catfish linkage map

**Figure 1:**
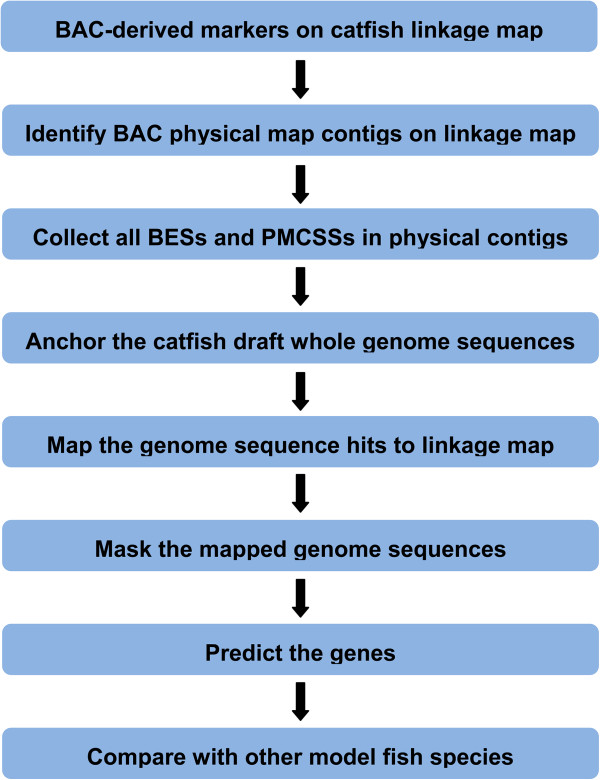
Flowchart for anchoring the draft genomic sequences on catfish linkage groups.

Comparative sequence analysis of species with fully sequenced genomes is relatively straight-forward. However, it is difficult for most teleost fish such as catfish whose whole genome sequence assembly is not yet available. In such cases, one of the key steps for genome-scale comparative analysis is to identify whole genome sequences that are anchored on the linkage map. For catfish, the sequence-tagged markers existing on the linkage map are the BAC-derived microsatellite markers [48]. Therefore, as shown in Figure 1, this study starts with BAC-derived microsatellites on the linkage map, followed by several steps utilizing available genome resources including the integrated genetic linkage and physical map [48], BAC end sequences [45,54], physical map contig-specific sequences (PMCSSs) [65], and the anchored catfish genome scaffolds (unpublished data, Table 1). A total of 2,099 BAC end sequence-derived microsatellite markers were mapped on the catfish linkage map ([48], Table 2). Based on these marker-associated BACs, a total of 931 physical map contigs were linked with the linkage map. A total of 32,500 available BAC end sequences were identified from the BAC clones of the 931 physical map contigs. In addition, a total of 57,861,110 physical map contig-specific sequences were identified ([65], Table 2). Both BAC end sequences and physical map contig-specific sequences were used to anchor the draft catfish whole genome sequences to catfish genetic linkage map. Taken together, a total of 517 Mb (62%) draft catfish genome sequences were anchored to the linkage map (Table 2). As described in another study [65], PMCSSs are short specific sequences randomly distributed in each catfish physical map contig, which can serve as anchor points to map the draft catfish genome sequences to the linkage group. Without PMCSSs, only 26% of the draft genome sequences can be map onto linkage group. A number of the draft genome sequences were stacked together due to the relatively low resolution of the catfish linkage map. In those cases, the gene orders and orientations were not resolved, though the relatively large-scale chromosome locations were known when examined at a “zoomed out” fashion.

**Table 1:** Summary of statistics of anchored scaffolds

No. of scaffolds	62,461
N50 of scaffolds (bp)	3,016,365
Total span (Mb)	832
No. of the anchored scaffolds	41,061
N50 of the anchored scaffolds (bp)	1,935,203
Total length of the anchored scaffolds (Mb)	517

**Table 2:** Summary of genome resources used to anchor genes to catfish linkage groups

**Category**	**Number**
BAC-associated markers in linkage map	2,099
BAC contigs containing the BAC-associated markers	931
All BAC-end sequences (BES) from mapped BAC contigs	32,500
Physical map contig-specific reads (PMCSS)	57,861,110
Total length of mapped draft genome contigs (Mb)	517 (62%)
Unique medaka genes with mapped genome contig hits	9,949
Medaka gene hits mapped to chromosomes	9,036
Unique Tetraodon genes with mapped genome contig hits	9,920
Tetraodon gene hits mapped to chromosomes	7,181
Unique stickleback genes with mapped genome contig hits	10,430
Stickleback gene hits mapped to chromosomes	9,465
Unique zebrafish genes with mapped genome contig hits	14,035
Zebrafish gene hits mapped to chromosomes	13,784

### Identification of homologous genes

The 517 Mb genome sequences retrieved from the draft catfish genome scaffolds were used for further comparative genome analysis. Genes located in these sequences were identified by BLASTX search against ENSEMBL protein database, including protein sequences from zebrafish, medaka, *Tetraodon*, and stickleback. Homologous genes in these species were identified as summarized in Table 2. The largest number of homologous genes (14,035) was found in zebrafish genome. Of the 14,035 homologous genes, 13,784 genes have chromosome information based on current zebrafish genome annotation in ENSEMBL (Table 2). A total of 9,949 homologous genes were identified in medaka genome. Of which, 9,036 genes were mapped on the chromosomes of medaka genome. Similar numbers of homologous genes were identified from *Tetraodon* and stickleback genome, with 7,181 and 9,465 being mapped to the chromosomes, respectively (Table 2).

### Identification of homologous chromosomes

**Figure 2:**
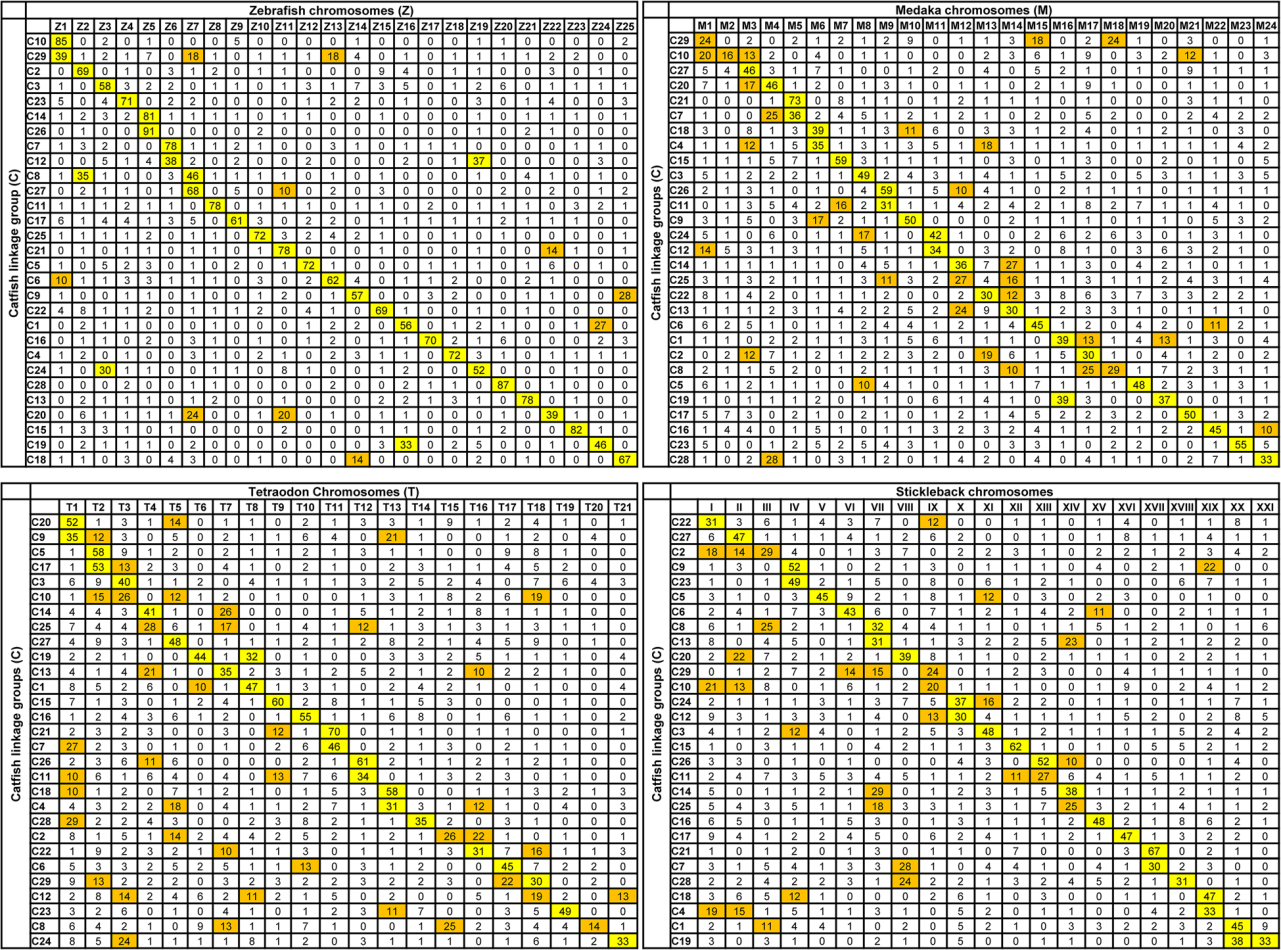
**Homologous chromosome relationships between catfish and four model fish species.** In each case, the catfish linkage group (LG) are displayed in rows, and the model fish species (zebrafish, medaka, stickleback, *Tetraodon*) chromosomes are displayed in columns. The Numbers in the cell is the percentage of homologous genes between catfish and other model fish species located on this chromosome (LG). Percentage higher than 30% is highlighted with yellow color, while percentage lower than 30% but higher than 10% is highlighted with orange color.

The identified homologous genes were used to determine the homologous chromosomes. All the genes identified for each of the catfish linkage group were used as queries to search against the protein sequences of the four model fish species. The homologous chromosomes for each catfish linkage group were determined based on the percentages of catfish genes that had significant hits with genes from the corresponding chromosomes of the species used for comparative analysis. For instance, 85% of the catfish genes in linkage group 10 had significant hits to genes located in zebrafish chromosome 1 (Figure 2), and therefore, catfish LG10 is the homologous chromosome of zebrafish chromosome 1. As shown in Figure 2, the relationships between catfish linkage groups and chromosomes of the four model fish species were established.

Eleven catfish linkage groups and zebrafish chromosomes had a one-to-one homologous relationship. These linkage groups included LG4, LG5, LG11, LG13, LG15, LG16, LG17, LG22, LG23, LG25 and LG28. Of all 29 catfish linkage groups, 17 linkage groups were found to be homologous to a single chromosome in zebrafish. Of the 17 catfish linkage groups, five are extremely highly conserved with over 81-91% of their genes shared between the catfish linkage groups and the zebrafish chromosomes. The catfish LG26 and zebrafish chromosome 5 shared 91% of the genes, followed by LG28 sharing 87% genes with zebrafish chromosome 20, LG10 sharing 85% genes with zebrafish chromosome 1, LG15 sharing 82% genes with zebrafish chromosome 23, and LG14 sharing 81% genes with zebrafish chromosome 5 (Figure 2).

Twelve catfish linkage groups were found to be homologous to more than one chromosome in zebrafish. Of which, 10 catfish LGs were homologous to two zebrafish chromosomes each, and two catfish LGs were homologous to three zebrafish chromosomes each (Figure 2). For instance, homologous genes located on catfish linkage group 12 were found in both zebrafish chromosome 6 (38%) and chromosome 19 (37%). Similarly, homologous genes located on catfish linkage group 29 were found in three zebrafish chromosomes: chromosome 1 (39%), chromosome 7 (18%), and chromosome 13 (18%).

When the vast majority of the genes located on one catfish linkage group are homologous to genes located on a single zebrafish chromosome, e.g. catfish LG26 and zebrafish chromosome 5 that share 91% of the genes, it is apparent that these chromosomes are homologous chromosomes. However, when much lower percentage of genes are homologous between a catfish linkage group and a zebrafish chromosome, e.g., around 10%, further analysis is required to provide information as to if chromosomal segments are orthologous with conserved syntenies. Examination of genes and their orders within the catfish scaffolds and zebrafish chromosomes demonstrates that they are indeed syntenic and therefore, likely orthologous. For instance, 27% of genes on catfish LG1 are homologous to genes on zebrafish chromosome 24. On zebrafish chromosomes, these genes were organized in two genomic segments, one spanning approximately 16 Mb from the beginning of the chromosome 24 (position 2,096) to position 16,741,284, and the other spanning approximately 14.5 Mb starting from position 29,305,847 to position 43,867,471 (Additional file 1). In catfish, as the whole genome assembly is not yet available, our analysis is limited to locate multiple genes within a single scaffold, followed by the analysis of the physical map and linkage map positions of the involved scaffolds. As shown in Additional file 1, many genes located in the same zebrafish genomic segments were also located in a single scaffold of the catfish draft genome sequence, and these scaffolds were mapped to similar locations on the linkage map.

Chromosome level conservation was the highest between catfish and zebrafish followed by stickleback, medaka, and *Tetraodon*. As shown in Figure 2, the one-to-one chromosome relationship was also observed between catfish and medaka, catfish and *Tetraodon*, and catfish and stickleback, but apparently at a lower level as compared with the situation between catfish and zebrafish. This was reflected at two levels. First, the percentage of homologous genes with a one-to-one relationship was much lower between catfish and medaka, catfish and *Tetraodon*, and catfish and stickleback as compared to catfish and zebrafish. Second, the level of chromosome rearrangements was much greater between catfish and medaka, catfish and *Tetraodon*, and catfish and stickleback as compared with catfish and zebrafish (Figure 2). The lowest level of chromosomal conservation was between catfish and *Tetraodon*.

### Identification of conserved syntenic blocks between catfish and zebrafish

Conserved synteny, a block of genes in certain chromosome region in which the content and order are relatively conserved, is an interesting phenomenon in genome evolution [66]. Establishing the conserved syntenies by conducting comparative analysis between species is not only valuable for genome assembly and annotation, but also valuable for functional and evolutionary genomics studies such as gene duplication and chromosome rearrangement [4,67]. To gain a close insight into the conserved chromosomal segments, conserved syntenies were examined between catfish and zebrafish in this study. As shown in Table 3, a total of 1,943 conserved syntenic blocks were identified, spanning approximately 703 Mb. The average size of the conserved synteny is 362 Kb. A total of 10,876 homologous genes were involved, with an average of 6 genes per syntenic block. For each catfish linkage group, the number of conserved syntenic blocks ranged from 32 to 105, spanning genomic region from 6 Mb to 39 Mb, with the number of genes varying from 126 to 577 (Table 3). In most cases, the number of syntenic blocks, spanning lengths, and the number of genes located in these syntenic blocks were highly correlated. The more highly conserved chromosomes (linkage groups) tend to have greater number of syntenic blocks identified, with extended spanning lengths involved in a larger number of genes (Table 3). Detailed information for the identified syntenic blocks is provided in Additional file 2.

**Table 3:** Summary of conserved syntenic blocks between catfish linkage groups and zebrafish chromosomes

**Group**	**Total length spanned (Kb)**	**No. of syntenies**	**Ave. size of the synteny (Kb)**	**Max. size of the synteny (Kb)**	**N50 of the synteny (Kb)**	**No. of genes involved**	**Ave. gene number/synteny**
LG1	39,522	105	361	1,997	1,276	577	6
LG2	11,105	54	206	942	340	202	5
LG3	26,165	93	281	1,475	519	439	5
LG4	21,282	65	327	2,091	854	335	6
LG5	36,292	80	454	3,189	964	503	8
LG6	36,722	102	360	2,835	810	570	7
LG7	21,884	48	456	2,341	984	319	8
LG8	26,601	92	289	3,269	631	434	5
LG9	27,314	66	414	3,702	932	431	8
LG10	15,589	41	380	3,247	709	277	9
LG11	26,953	72	374	4,995	1,121	475	8
LG12	22,483	79	285	2,731	521	389	6
LG13	29,613	78	380	2,363	707	472	8
LG14	13,468	43	313	2,631	749	231	6
LG15	27,758	78	356	2,466	632	453	8
LG16	36,037	105	343	1,803	684	535	5
LG17	35,452	92	385	1,651	658	464	6
LG18	19,451	68	286	1,536	526	333	6
LG19	20,872	36	580	3,201	1,604	269	9
LG20	31,788	73	435	3,800	844	456	9
LG21	23,200	56	414	3,730	835	315	6
LG22	28,266	69	410	2,233	842	431	7
LG23	11,486	47	244	977	465	221	5
LG24	24,625	46	535	2,529	1,075	363	10
LG25	23,276	79	295	1,539	759	407	6
LG26	18,055	46	393	3,541	803	256	7
LG27	15,045	35	430	2,465	1,103	204	7
LG28	26,602	63	422	2,382	1,010	389	8
LG29	5,860	32	183	705	345	126	5
Total	702,766	1,943				10,876	

### Chromosomal level structural conservations

To gain detailed understanding of evolutionary relationship between catfish and zebrafish, a comparative map was constructed between the catfish linkage groups with their homologous chromosomes in zebrafish. Only gene sequences were used for this comparative analysis because gene sequences are more conserved than sequences in intergenic regions. The positions of physical map contigs were determined in the linkage group based on the locations of BES-associated markers. However, the positions and orders of genes within each physical contig cannot be determined because of the incompletely assembled genome sequences. In addition, a number of catfish genes were stacked because of the low resolution provided by the current genetic linkage map.

**Figure 3:**
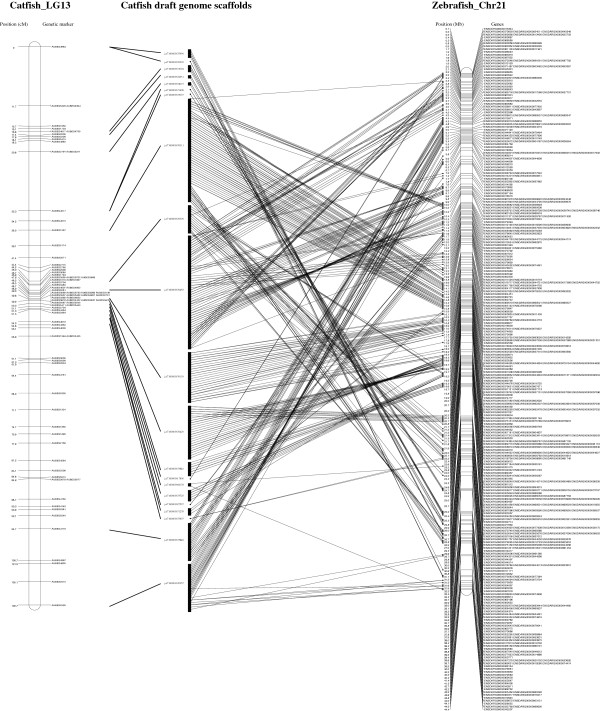
**Comparative map of catfish LG13 and zebrafish chromosome 21.** The catfish LG13 is presented on the left panel while the zebrafish chromosome 21 is on the right panel. The gene-associated catfish draft genome contigs (scaffolds) is presented in the middle panel. For catfish LG13, genetic linkage position is indicated in cM on the left of the bar, and the BAC-derived microsatellite markers are indicated on the right of the bar. For zebrafish chromosome 21, gene locations along the chromosome are indicated in Mb on the left while gene names are indicated on the right.

A comparative map of catfish linkage group 13 to the corresponding zebrafish chromosome 21 is shown in Figure 3. A total of 447 genes on catfish LG13 spanneda genetic distance 109 cM [48]. The homologous genes of the 447 genes on catfish LG13 are distributed almost across the entire zebrafish chromosome 21, from position 64 Kb to 44,353 Kb (Figure 3). The results indicated a high level of genome-level conservation existing between the catfish linkage group and the zebrafish chromosome. However, as shown in Figure 3, numerous chromosome rearrangements were involved during evolution.

Similar results were observed in the other 28 catfish linkage groups, with general large scale chromosome level of genome conservation, but with numerous chromosome breaks, shuffling and rearrangements, which are consistent with previous studies [46,51].

## Discussion

Rapid development of genomic resources in fish species has provided the opportunity for comparative genome analysis, shedding lights on the structure, organization, function and evolution of vertebrate genomes. In this study, we conducted the whole genome comparative analysis of channel catfish, an important aquaculture species, with several model fish species. By comparing with other fully sequenced model fish species including zebrafish, medaka, stickleback, and *Tetraodon*, homologous chromosomes among these species were determined and a large number of conserved syntenies were identified, providing valuable information for whole genome assembly and annotation in catfish, and for comparative genome analysis of other teleost species.

Comparative map is a powerful tool in genomics studies, especially for non-model species, by transferring the genomic information from well-studied model species. Comparative map not only allows better understanding genome arrangement during evolution, but also benefits the discovery or confirmation of orthologies among species.

Without a well assembled whole genome sequences, comparative genome analysis can be achieved by using various other genomic resources containing information for genome level conservation. Markers with low levels of conservation in evolution have very limited value for comparative genome analysis, and most often only for very closely related species. For instance, when microsatellite markers on linkage maps were used for comparative analysis, a small number of microsatellites could be successfully mapped, indicating relative low levels of conservation of microsatellites which were derived from non-coding regions of the genome [68]. Comparative analysis among different species using gene-derived markers on linkage maps was more effective because genes are well conserved through evolution [46]. Higher resolution of comparative maps can be achieved by using integrated physical and genetic linkage maps with BAC end sequences. For instance, Zhang et al. [64] conducted comparative analysis of one catfish chromosome (linkage group 8) with four model fish species utilizing catfish linkage map, physical map, BAC end sequences and draft genome sequences. In that work, 287 unique genes were identified, and a number of conserved syntenies were identified. Although that work demonstrated the utilities of linkage maps when integrated with physical maps with BAC end sequences, the ability to establish whole genome comparative map was hindered by the lack of internal BAC sequences. In order to increase the power of comparative genome analysis, we recently developed one additional valuable genome resource, the physical map contig-specific sequences [65]. In this study, we used all the existing genome resources of catfish for whole genome comparative analysis. The ability to identify long conserved syntenic blocks was much enhanced. For instance, with the same linkage group, LG8, as used in Zhang et al. [64], we were able to identify 585 unique genes, more than the double of the 287 genes identified in the previous study [64], and the size of syntenic blocks were much increased.

The strategy developed in this study allowed whole genome comparative analysis of catfish conducted without a well-assembled whole genome sequence. However, lacking of a continuous reference sequences, the order and orientation of catfish draft genome contigs/scaffolds within the same catfish physical contig cannot be determined at present. The reason for this inability is the low resolution of the linkage map. Because of the low resolution of the linkage map, many genes are mapped directly or indirectly to the same map location, forming stacks of sequence contigs and scaffolds with gene orders and orientations undetermined. Therefore, it is imperative to develop linkage maps with many more markers or using high density marker such as SNPs in the future, and more importantly with high resolution by using large resource families.

Comparative analysis was conducted based on the similarities between gene sequences in catfish and the homologous genes in the genomes of other model fish species. Only genes were used for analysis in this study because gene sequences are more conserved than intergenic sequences. The largest number of homologous genes was found in zebrafish among four model fish species, with 14,035 genes, followed by stickleback with 10,430 genes, medaka with 9,949 genes and *Tetraodon* with 9,920 genes. This difference of the homologous genes identified in different model fish species may be resulted from two reasons: First, the quality of the reference genome. For instance, of all annotated 32,574 zebrafish genes, only 1,225 (3.7%) are unmapped onto zebrafish chromosomes or mitochrondria, while in *Tetraodon*, there are 6,487 (31.5%) of all 20,562 genes cannot be mapped (Ensembl database). Second, the phylogenetic relationship between catfish and these model fish species determined that zebrafish is the most closely species to catfish [63,69-71].

High levels of chromosomal conservations were observed between catfish and the four fish species. However, due to the difference of chromosome numbers among those fish species, e.g. catfish has 29 chromosomes, while zebrafish has 25 chromosomes, *Tetraodon* has 21 chromosomes, medaka has 24 chromosomes and stickleback has 21 chromosomes, chromosome breakage or fusion would have occurred during evolution. For instance, 33% of genes identified on catfish LG19 were found to be homologous in zebrafish chr.16, and 46% of genes were found to be homologous in zebrafish chr.24 (Figure 2), suggesting catfish LG19 have been created by fusion of chromosomal segments similar to zebrafish chr.16 and chr.24, or inversely the two zebrafish chromosomes have been created by split of the chromosome similar to catfish LG19. Similar cases can be observed between the comparison of catfish and medaka, catfish and *Tetraodon*, catfish and stickleback, indicated that chromosomal fusions or splits occurred frequently during the teleost evolution.

Sarropoulou et al. [7] conducted a comparative study in which the syntenic relationship between six non-model fish species genomes were established by using ESTs and microsatellites sequences. Our study here extended that study by adopting a much larger numbers of genes. For instance, catfish LG15 was identified to be the homologous chromosome of medaka chr.7 (C7), *Tetradon* chr.9 (T9), and stickleback Grp. XII (SXII), respectively, which indicated that C7, T9 and SXII were homologous chromosomes to one another. This was consistent with the results of Sarropulou et al. [7]. Similarly, catfish LG9 (C9) corresponded to *Tetraodon* T1, T2 and T13 in this study, while T1 was reported to be homologous chromosome to medaka M10 [7], which also had the highest percentage homologous gene hits to catfish LG9 in our study.

Because zebrafish is the most closely related model fish to catfish, detailed comparative analyses were conducted between them. Catfish has 29 pairs of chromosomes while zebrafish has 25 pairs of chromosomes. Therefore, some zebrafish chromosomes are expected to be homologous to greater than one chromosome in catfish. This was found with several chromosomes (Figure 2). However, the opposite situation was also found with one catfish chromosome being homologous to several zebrafish chromosomes (Figure 2), suggesting extensive chromosome rearrangements during evolution.

A large number of conserved syntenic blocks between catfish and zebrafish were established. Analysis of the conserved syntenies should greatly benefit genome annotation in catfish. This is particularly true when dealing with large gene families and duplicated genes. As reported by Liu et al. [39], identities of genes involved in large gene families such as the ABC transporter gene families sometimes cannot be resolved by phylogenetic analysis alone. Syntenic analysis is essential to provide orthologous information for the identification of such genes. The inferred orthologies are important not only for the identification and annotation of genes, but also for functional inference based on orthologies [64]. Apparently, catfish genome is well conserved at the chromosomal level with those in other model fish species. However, local chromosome shuffling and rearrangements are extensive (Figure 3). Our whole genome comparative analysis with four teleost species also indicated extensive inter-chromosomal rearrangements during evolution, consistent with the hypothesis that inter-chromosomal rearrangements were increased after whole genome duplication in the teleost lineage [67,72].

## Conclusions

Whole genome comparative analysis of channel catfish was conducted by utilizing currently available catfish genomic resources including genetic linkage map, physical map, BAC end sequences, physical map contig-specific sequences, and the draft whole genome sequences. Homologous genes and homologous chromosomes of catfish as compared with four fully sequenced fish species were identified based on sequence similarities and arrangements of homologous genes along the chromosomes. Detailed comparative analysis between catfish and zebrafish allowed for the establishment of a large number of conserved syntenies, with some being extended in large sizes. The whole genome comparative analysis should facilitate whole genome sequence assembly and annotation, as well as providing insight into genome evolution.

## Methods

### Anchorage of draft genomic sequences on catfish linkage groups

Various currently available catfish genome resources were utilized in this study, including the genetic linkage map [48], BAC-derived microsatellite markers [48], BAC-based physical map [53], BAC end sequences [45,54], and draft genome sequences (Unpublished data). As shown in Figure 1, the steps to anchor draft genomic contigs on catfish linkage groups are: 1) Starting with all BAC-derived microsatellite markers on the catfish linkage map; 2) Using the markers to identify BAC end sequences from which they were derived; 3) Using the BAC end sequences to determine the physical map contigs mapped on the linkage groups; 4) Collecting all BAC end sequences and the physical map contig-specific sequences from all mapped physical contigs; 5) Using the assembly of BAC end sequences and physical map contig-specific sequences to search for corresponding whole genome draft sequence contigs and scaffolds using BLAST. All identified draft genome sequences and their respective chromosome (linkage group) locations were used for further comparative analysis.

### Identification of homologous genes

Before the gene identification, RepeatMasker (Version 3.2.7, http://www.repeatmasker.org/) was used to mask repetitive-elements within the draft genome sequences. The repeat-masked sequences were then used as query for BLASTX searches against the ENSEMBL protein database of other fully sequenced model fish species, including zebrafish (*Danio rerio*), medaka (*Oryzias latipes*), stickleback (*Gasterosteus aculeatus*) and *Tetraodon* (*Tetraodon nigroviridis*), with an E-value cutoff of 1e-10. Gene annotation information was retrieved using BioMart (http://www.ensembl.org/biomart/martview) with ENSEMBL protein ID.

### Identification of homologous chromosomes

The genome locations of homologous genes from zebrafish, medaka, stickleback and *Tetraodon* were obtained by using BioMart with respective ENSEMBL gene IDs. The homologous chromosomes corresponding to each catfish linkage group (chromosome) from zebrafish, medaka, *Tetraodon* and stickleback were determined as the chromosome with a majority of homologous genes. In cases where significant fractions (more than 10%) of genes were located on several chromosomes, all these chromosomes were determined to contain homologous genomic segments.

### Identification of conserved syntenies

Conserved syntenies were defined as preserved co-localization of genes on chromosomes from different species. Conserved syntenies were identified based on the genetic locations of BAC-derived microsatellite markers and their associated genes on the linkage map and the genome positions of homologous genes from other model fish species. In this study, conserved syntenies were established when at least two adjacent genes on the model fish chromosome were found within a single contig of the draft catfish genome or scaffold.

Comparative maps between each catfish linkage group with the homologous chromosome from zebrafish were conducted by using MapChart [73]. The genes within a physical map contig were located on catfish linkage group based on the position of BAC-derived microsatellite markers [48]. The comparative maps were drawn based the gene position on catfish linkage map and the position of their homologous genes from zebrafish chromosomes.

## Competing interests

The authors declare that they have no competing interests.

## Authors’ contributions

YJ conducted the major part of the project including data analysis and manuscript preparation. XG, SL, YZ, HL, FS and LB assisted in data analysis, GW involved in generation of catfish genome sequence resources used in this study. ZL designed and supervised the project, and finalized manuscript. All authors read and approved the manuscript.

## Supplementary Material

Additional file 1 **Homologous chromosome relationships with homologous genes between catfish and zebrafish.** The conserved syntenic blocks are highlighted. Click here for file

Additional file 2 **The detail information of conserved syntenic blocks identified between catfish and zebrafish.** The conserved syntenic blocks are highlighted. Click here for file
